# Effects of Conventional and Ozonated Autohemotherapy as Adjuvant Treatment in Dogs with Transmissible Venereal Tumor (TVT)

**DOI:** 10.3390/ani16121913

**Published:** 2026-06-20

**Authors:** Neusvaldo de Medeiros Caldas Júnior, André Sampaio Calheiros, Keityane de Oliveira e Silva, Danillo de Souza Pimentel, Márcia Kikuyo Notomi, Pierre Barnabé Escodro

**Affiliations:** Research and Extension Group on Equines and Integrative Health, Federal University of Alagoas, Viçosa 5770000, AL, Brazil; nmcjr.28@gmail.com (N.d.M.C.J.); andresampaiocalheiros@gmail.com (A.S.C.); danillo.pimentel@vicosa.ufal.br (D.d.S.P.); marcia.notomi@vicosa.ufal.br (M.K.N.); pierre.vet@gmail.com (P.B.E.)

**Keywords:** sticker tumor, chemotherapy, autohemotherapy, blood

## Abstract

Transmissible venereal tumor (TVT) is a contagious neoplasm affecting dogs, commonly treated with vincristine sulfate, although this protocol may be associated with adverse effects and the need for multiple therapeutic sessions. In this context, adjuvant therapies have been investigated to optimize clinical outcomes. This study evaluated the effect of conventional and ozonated autohemotherapy on the number of chemotherapy sessions required to achieve complete remission of TVT. Fifteen dogs with a confirmed diagnosis were randomly allocated into three groups: control (vincristine, *n* = 5), conventional autohemotherapy associated with vincristine (AHTm, *n* = 5), and ozonated autohemotherapy associated with vincristine (AHTmO_3_, *n* = 5). The animals were evaluated weekly through clinical, cytological, hematological, and biochemical examinations. The primary outcome was the number of sessions required to achieve complete remission. A significant reduction was observed in the AHTm (*p* = 0.032) and AHTmO_3_ (*p* = 0.008) groups compared to the control group. No differences were found in laboratory parameters. The findings suggest that autohemotherapy, particularly in its ozonated form, may serve as a promising adjuvant strategy. However, studies with larger sample sizes are needed.

## Simple Summary

The Transmissible venereal tumor (TVT) is a contagious neoplasm affecting dogs, typically treated with vincristine-based chemotherapy. Although effective, this treatment may require multiple sessions and can cause adverse effects. This study investigated whether autohemotherapy, a technique that uses the animal’s own blood to stimulate the immune system, could improve treatment outcomes. In addition, the association of this technique with ozone therapy was evaluated, given its biological properties that may enhance the organism’s response. The results showed that dogs receiving these combined therapies required fewer chemotherapy sessions to achieve complete remission, without an increase in adverse effects. These findings suggest that these approaches may contribute to a faster and potentially more comfortable treatment for the animals. However, further studies are needed to confirm these results.

## 1. Introduction

Transmissible venereal tumor (TVT) is a round cell neoplasm of mesenchymal origin, characterized by its contagious nature and transmission predominantly through direct tissue contact, especially during coitus, although extragenital forms have also been described [1,2]. The disease has a worldwide distribution, with higher prevalence in tropical and subtropical regions, and is considered an important health problem in canine populations, particularly among free-roaming animals [3,4,5].

TVT is currently recognized as a neoplasm of histiocytic origin, typically expressing vimentin and CD18, while lacking lymphoid markers such as CD3 and CD79a [6]. This immunophenotypic profile reinforces its non-lymphoid nature, despite the cytomorphological variability observed in tumor cells [7,8].

The diagnosis of TVT is based on clinical evaluation associated with cytological examinations, such as imprint cytology and fine-needle aspiration cytology (CAAF), and may be confirmed by histopathological examination when necessary [1,9,10]. Cytomorphological classification into lymphocytoid, plasmacytoid, and mixed (lymphoplasmacytoid) patterns has been associated with differences in biological behavior and treatment response, with the plasmacytoid pattern often related to lower sensitivity to chemotherapy [8,11]. However, these classifications represent morphological variants of the same neoplastic population and do not indicate true lymphoid or plasma cell differentiation.

Chemotherapy with vincristine sulfate is considered the treatment of choice for TVT, showing high remission rates, including in metastatic cases [7,12]. However, the therapeutic protocol may require multiple weekly sessions and is associated with adverse effects, such as hematological alterations and gastrointestinal disorders, which may impact animal welfare and treatment adherence [13].

In this context, adjuvant therapies have been investigated with the aim of enhancing therapeutic response, reducing treatment duration, and minimizing adverse effects. Autohemotherapy has been proposed as a nonspecific immunomodulatory approach, based on the reinfusion of autologous blood to stimulate the immune response [14]. However, its mechanisms of action are not fully elucidated, and its clinical efficacy remains a subject of debate due to the limited number of controlled studies and the lack of standardized protocols.

Similarly, ozone therapy has attracted interest due to its potential biological effects, including modulation of oxidative stress, improvement of tissue oxygenation, and stimulation of the immune response [15,16]. These effects are attributed to the controlled induction of oxidative processes capable of triggering adaptive cellular responses [15,16,17]. In veterinary medicine, ozonated autohemotherapy, in which blood is exposed to ozone prior to reinfusion, has been investigated as a complementary approach in different clinical conditions; however, evidence in oncological contexts remains limited [17,18,19,20,21,22].

Despite the increasing use of these integrative therapies, there is still a lack of controlled studies evaluating their efficacy as adjuvant treatment in canine TVT. Therefore, the present study aimed to provide initial clinical evidence regarding the effect of conventional autohemotherapy and ozonated autohemotherapy on the number of chemotherapy sessions required to achieve complete remission in dogs with TVT treated with vincristine sulfate.

## 2. Materials and Methods

### 2.1. Study Design and Ethical Considerations

This is a prospective experimental study with a quantitative approach, conducted in dogs naturally affected by transmissible venereal tumor (TVT). The study was approved by the Institutional Animal Care and Use Committee (CEUA) of the originating institution, under protocol no. 03/2023, and was carried out with the informed consent of the animals’ owners.

### 2.2. Animals and Inclusion Criteria

Fifteen intact mixed-breed dogs with a confirmed diagnosis of transmissible venereal tumor (TVT) were included in the study. As inclusion criteria, animals were required to present lesions clinically compatible with TVT and not to have been previously subjected to chemotherapy. Animals with severe comorbidities or clinical conditions that contraindicated treatment were excluded from the study.

Prior to inclusion, all animals underwent clinical examination, complete blood count, serum biochemical analysis, and screening for vector-borne infectious diseases using the SNAP 4Dx^®^ test (IDEXX Laboratories, Westbrook, ME, USA). Only animals considered clinically eligible for the proposed protocol were enrolled.

The diagnosis of TVT was confirmed by cytological evaluation of impression smears (imprint cytology) obtained directly from the lesions. For each animal, three cytological slides were prepared. Two slides were submitted to the Clinical Pathology Laboratory of the Veterinary Teaching Hospital of the Federal University of Alagoas (UFAL), and one slide was submitted to a private veterinary diagnostic laboratory. Cytological diagnosis and cytomorphological classification were performed by experienced veterinary clinical pathologists according to previously established criteria for TVT classification. Tumors were classified as lymphocytoid, plasmacytoid, or lymphoplasmacytoid based on their predominant cytomorphological characteristics.

At enrollment, all animals presented preserved general clinical condition and body condition scores ranging from 3 to 4 on a 5-point scale, with no significant weight loss or clinical abnormalities that contraindicated participation in the study.

### 2.3. Experimental Allocation

The animals were randomly allocated into three experimental groups (*n* = 5 per group) through simple randomization performed prior to the initiation of treatment:

- Control group (CG): treated with vincristine sulfate;
- AHTm group: minor autohemotherapy associated with vincristine sulfate;
- AHTmO_3_ group: ozonated minor autohemotherapy associated with vincristine sulfate.

### 2.4. Therapeutic Protocol

Vincristine sulfate was administered intravenously at a dose equivalent to 0.75 mg/m^2^ of body surface area at weekly intervals until complete remission of the lesions.

In the AHTm and AHTmO_3_ groups, minor autohemotherapy was performed approximately 10 min prior to chemotherapy administration. For this purpose, venous blood was collected at a volume of 0.5 mL/kg using aseptic technique and immediately reinfused intramuscularly into the semimembranosus muscles.

In the AHTmO_3_ group, the collected blood was previously subjected to ozonation at a 1:1 volumetric ratio with an ozone/oxygen gas mixture at a concentration of 32 µg/mL, using a medical-grade ozone generator. Ozonation was performed immediately after collection, with gentle homogenization of the blood prior to reinfusion.

The ozone concentration of 32 µg/mL was selected based on concentrations previously described in ozone therapy protocols and recommendations aimed at promoting biological modulation while minimizing excessive oxidative damage, in accordance with the controlled oxidative and adaptive responses described for ozone therapy [17,18,21].

During the procedures, all animals received fluid therapy with lactated Ringer’s solution (10 mL/kg), and vincristine was administered as a bolus at an approximate rate of 2 mL/min.

### 2.5. Clinical and Laboratory Evaluation

The animals were evaluated weekly, with each evaluation defined as a weekly time point (WT), beginning at baseline (WT1).

At each evaluation, a complete clinical examination was performed, including heart rate, respiratory rate, body temperature, and body weight. Tumor lesions were assessed for size, number, location (genital or extragenital), and presence of metastases.

Diagnostic confirmation and monitoring of tumor regression were performed using imprint cytology. Cytological evaluation was performed using rapid panoptic staining (Renylab^®^, Barbacena, MG, Brazil), chosen for its rapid execution, practicality, and suitability for routine veterinary cytology.

Blood samples were collected for complete blood count and serum biochemical analysis, including alanine aminotransferase (ALT), creatinine, alkaline phosphatase, and urea.

### 2.6. Experimental Outcome

The primary outcome of the study was the number of chemotherapy sessions required to achieve complete remission, defined as the absence of macroscopic tumor lesions associated with negative cytology.

After remission, an additional chemotherapy session was administered to all animals. The dogs were followed for a period of 90 days to monitor for possible recurrence.

### 2.7. Statistical Analysis

Data were analyzed using SPSS software (version 13.0, IBM Corp., Armonk, NY, USA). Initially, descriptive analysis of the variables was performed, including calculation of the mean, standard deviation, and minimum and maximum values.

Data normality was assessed using the Shapiro–Wilk test. For analysis of the primary outcome (number of sessions until remission), one-way analysis of variance (ANOVA) was used, followed by Tukey’s post hoc test for multiple comparisons when normality assumptions were met. For nonparametric variables, the Kruskal–Wallis test was applied, followed by the Mann–Whitney test for pairwise group comparisons. The level of significance adopted was 5% (*p* < 0.05).

## 3. Results

Fifteen dogs with a confirmed diagnosis of transmissible venereal tumor (TVT) were evaluated, of which 73.3% (11/15) were females and 26.7% (4/15) were males. Most lesions were located in the genital region (86.7%; 13/15), while one case (6.7%) was extragenital and another (6.7%) showed concurrent genital and extragenital involvement.

Clinically, the main findings included serosanguineous to sanguineous discharge and swelling in the affected region. According to the owners’ reports, the mean duration of the lesions was approximately three months.

Regarding tumor size, 6.7% (1/15) of the animals presented lesions smaller than 1 cm, 40.0% (6/15) between 1 and 5 cm, and 53.3% (8/15) larger than 5 cm in diameter.

**Figure 1:**
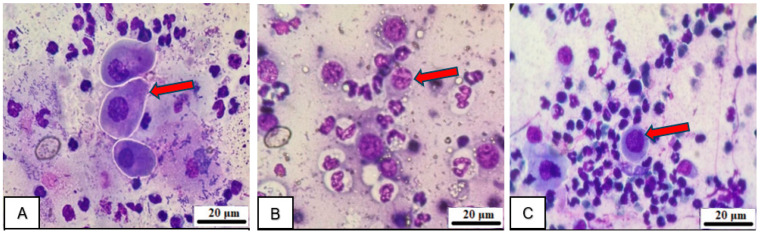
Representative cytological images of transmissible venereal tumor (TVT) in dogs showing different cytomorphological patterns: (**A**) plasmacytoid morphology; (**B**) lymphocytoid morphology; (**C**) mixed (lymphoplasmacytoid) morphology. Arrows indicate neoplastic TVT cells. Rapid panoptic stain. Scale bar: 20 µm.

Cytomorphological classification revealed a predominance of the lymphocytic (46.7%; 7/15) and lymphoplasmacytic (46.7%; 7/15) subtypes, with only one case of the plasmacytic type (6.7%) (Figure 1).

During treatment, five animals (33.3%) exhibited mild apathy associated with partial anorexia in the first days following chemotherapy administration. Episodes of vomiting were observed in three animals (20.0%) after the first session, and one animal (6.7%) developed phlebitis. All adverse effects were considered mild and self-limiting.

No statistically significant differences were observed among the groups regarding the evaluated physiological parameters, including heart rate, respiratory rate, body temperature, and body weight throughout the treatment.

The number of sessions required to achieve complete remission differed among the experimental groups (Table 1). The control group (CG) showed a mean of 5.4 ± 0.89 sessions, whereas the AHTm group had a mean of 3.6 ± 0.89 sessions, and the AHTmO_3_ group had a mean of 2.4 ± 0.55 sessions.

**Figure 2:**
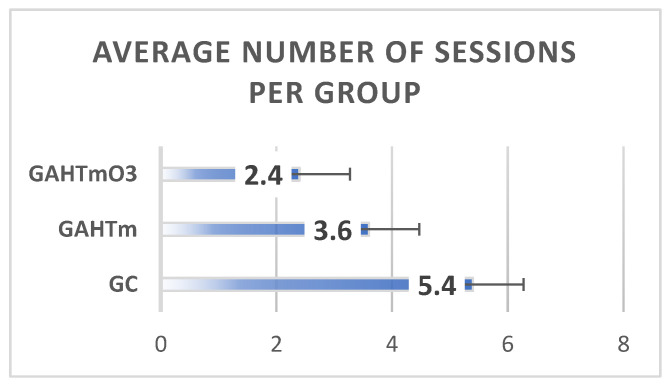
Number of sessions by treatment group: CG, Control Group; AHTm, Minor Autohemotherapy; AHTmO_3_, Ozonated Minor Autohemotherapy.

Statistical analysis demonstrated a significant reduction in the number of sessions in the AHTm (*p* = 0.032) and AHTmO_3_ (*p* = 0.008) groups compared to the control group. No statistically significant difference was observed between the AHTm and AHTmO_3_ groups (*p* = 0.056) (Figure 2).

Regardless of the treatment protocol or cytomorphological subtype, all animals achieved complete remission, and no cases of resistance to vincristine sulfate were observed.

The hematological and biochemical parameters evaluated between baseline (WT1) and the subsequent time point (WT2) showed no statistically significant differences among the groups (Table 2).

## 4. Discussion

Since the sample was selected according to predefined inclusion criteria rather than through an epidemiological design, the observed distribution of sex and lesion location should be interpreted descriptively. Females represented most of the evaluated animals, a finding that has also been reported in previous studies and is commonly associated with reproductive behavior during estrus, which may favor tumor transmission through increased exposure to multiple partners [24,25,26]. In addition, anatomical and hormonal factors may contribute to greater susceptibility to tumor cell implantation [15,25].

Most lesions were located in the genital region, consistent with the classical presentation of TVT described in the literature [15,25]. Although extragenital forms have been reported, they remain less frequent [9,10].

The variability in lesion size reinforces the clinical heterogeneity of TVT, which may present as single nodules, multiple nodules, or multilobulated masses, with wide variation in size, as previously described [27,28]. In addition to the therapeutic protocol, factors such as tumor size and cytomorphological subtype may also influence the number of chemotherapy sessions required to achieve remission.

Regarding cytomorphological classification, a predominance of lymphocytoid and lymphoplasmacytoid patterns was observed, while the plasmacytoid subtype was less frequent. This finding is consistent with previous studies [8] and has clinical relevance, as the plasmacytoid subtype has been associated with greater tumor aggressiveness and reduced response to chemotherapy [7,8]. In the present study, the single animal classified as plasmacytoid required a higher number of sessions to achieve remission, which may support this association; however, the limited representation of this subtype prevents more robust conclusions.

Treatment with vincristine sulfate demonstrated high efficacy, with complete remission observed in all animals, corroborating its widespread use as the treatment of choice for TVT [7,12]. However, the number of sessions required varied among groups, suggesting that adjuvant therapies may influence treatment dynamics.

The reduction in the number of sessions observed in the groups receiving autohemotherapy, particularly in its ozonated form, suggests a potential beneficial effect of these approaches as complementary therapies. Autohemotherapy has been described as a nonspecific immunomodulatory stimulus, possibly acting through activation of inflammatory and immune mechanisms [14]. However, its mechanisms of action are not fully elucidated, and scientific evidence supporting its clinical efficacy remains limited.

One possible hypothesis for the findings observed in this study is related to the immunobiological characteristics of TVT. Unlike many other neoplasms, TVT is a transmissible tumor whose progression and regression are strongly associated with the host immune response. In this context, adjuvant therapies capable of modulating inflammatory and immunological pathways could potentially influence tumor behavior and responsiveness to chemotherapy. Autohemotherapy, particularly in its ozonated form, may contribute to the activation of innate immune mechanisms, modulation of cytokine release, and improvement of tissue oxygenation, thereby creating a biological environment that enhances the antitumoral effects of vincristine. Although these mechanisms remain incompletely understood, this hypothesis may help explain the reduction in the number of chemotherapy sessions observed in the treated groups.

Both autohemotherapy and ozone therapy remain subjects of debate in the scientific literature, mainly due to the limited number of controlled studies, heterogeneity of protocols, and incomplete understanding of their mechanisms of action. Therefore, the findings of the present study should be interpreted with caution.

In the group treated with ozonated autohemotherapy, the more pronounced reduction in the number of sessions may be related to the biological effects attributed to ozone, including modulation of oxidative stress, improvement of tissue oxygenation, and stimulation of the immune response [15,16,17]. These effects are thought to result from controlled oxidative processes capable of inducing adaptive cellular responses and enhancing antioxidant defense systems, which may contribute to a more favorable environment for tumor regression, as described in previous studies [15,16,17,18,21]. Nevertheless, evidence in veterinary oncology remains limited, and these findings should be interpreted with caution [17,18,19,20].

The observed adverse effects were mild and self-limiting, consisting mainly of apathy, anorexia, and episodes of vomiting, consistent with the safety profile described for vincristine [29,30,31]. The reduced exposure to chemotherapy in the groups receiving adjuvant therapies may have contributed to the lower frequency and intensity of these effects, although this relationship was not directly evaluated.

Hematological and biochemical parameters did not show significant alterations among the groups, suggesting that the association of therapies did not result in relevant systemic effects during the evaluated period. These findings are consistent with studies reporting laboratory stability during vincristine treatment under conventional protocols [31].

This study presents important limitations, including the small sample size (n = 15), which may limit the generalizability of the findings, as well as the low representation of the plasmacytoid subtype. Additional limitations include the absence of blinding, the lack of evaluation of immunological and oxidative stress markers, the absence of prior sample size calculation, and the heterogeneity of cytomorphological variants among the studied animals. Furthermore, the absence of immunological marker evaluation limits a more in-depth understanding of the mechanisms potentially involved in the observed responses.

Overall, the results suggest that the association between conventional chemotherapy and integrative therapies, particularly ozonated autohemotherapy, may represent a potential adjuvant approach in the treatment of TVT. However, these findings should be interpreted with caution, and further controlled studies with larger populations, standardized protocols, and additional biological parameters are necessary to confirm these observations and better elucidate their mechanisms of action.

## 5. Conclusions

The association of autohemotherapy, particularly in its ozonated form, with vincristine sulfate chemotherapy may represent a potential adjuvant approach in the treatment of transmissible venereal tumors in dogs, as it was associated with a reduction in the number of treatment sessions required to achieve complete remission.

However, these findings should be interpreted with caution due to the limitations of the present study, particularly the small sample size, the limited representation of cytomorphological subtypes, and the absence of mechanistic evaluations. Further controlled studies with larger populations, standardized protocols, and additional biological parameters are necessary to confirm these observations and better elucidate the mechanisms involved.

## Figures and Tables

**Table 1:** Individual clinical and tumor characteristics of dogs with transmissible venereal tumor (TVT) according to treatment group. Tumor size was categorized as <1 cm, 1–5 cm, and >5 cm. CG: control group; AHTm: minor autohemotherapy; AHTmO_3_: ozonated minor autohemotherapy.

ID Animals	Group	Sex	Age (Years)	TVT Subtype	Tumor Location	Tumor Size	Ulceration	Number of Sessions
1.1	CG	Female	5	Lymphocytoid	Genital	>5 cm	Yes	4
1.2	CG	Female	2	Lymphocytoid	Genital	1–5 cm	Yes	5
1.3	CG	Female	6	Lymphoplasmacytoid	Genital	>5 cm	Yes	6
1.4	CG	Female	3	Lymphoplasmacytoid	Genital	1–5 cm	Yes	6
1.5	CG	Male	3	Lymphocytoid	Genital + extragenital	>5 cm	Yes	6
2.1	AHTm	Female	9	Lymphoplasmacytoid	Genital	>5 cm	Yes	3
2.2	AHTm	Female	4	Lymphoplasmacytoid	Genital	>5 cm	Yes	3
2.3	AHTm	Male	1	Lymphocytoid	Genital	1–5 cm	Yes	4
2.4	AHTm	Male	2	Plasmacytoid	Genital	1–5 cm	Yes	5
2.5	AHTm	Male	1	Lymphocytoid	Extragenital	<1 cm	Yes	3
3.1	AHTmO_3_	Female	4	Lymphocytoid	Genital	>5 cm	Yes	2
3.2	AHTmO_3_	Female	2	Lymphoplasmacytoid	Genital	>5 cm	Yes	3
3.3	AHTmO_3_	Female	3	Lymphocytoid	Genital	>5 cm	Yes	2
3.4	AHTmO_3_	Female	2	Lymphoplasmacytoid	Genital	1–5 cm	Yes	2
3.5	AHTmO_3_	Female	3	Lymphoplasmacytoid	Genital	1–5 cm	Yes	3

**Table 2:** Hematological and biochemical variables between WT1 and WT2 among the CG, AHTm, and AHTmO_3_ groups.

VARIABLE	WEEKLY TIME POINT 1			WEEKLY TIME POINT 2		
	CG	GAHTm	GAHTmO^3^	GC	GAHTm	GAHTmO3
Erythrocytes	4.86 ± 1.16 ^a^	5.68 ± 0.86 ^a^	4,73 ± 1.39 ^a^	4,61 ± 0.82 ^a^	4.79 ± 0.79 ^a^	5,08 ± 1.16 ^a^
Hemoglobin	10. 64 ± 3.10 ^a^	12.36 ± 1.79 ^a^	10.94 ± 3.23 ^a^	9.78 ± 3.08 ^a^	11.62 ± 2.65 ^a^	10.67 ± 2.48 ^a^
Hematocrit	32.60 ± 8.30 ^a^	38.20 ± 5.63 ^a^	32.00 ± 7.86 ^a^	31.60 ± 4.78 ^a^	33.80 ± 4.87 ^a^	34.00 ± 7.71
Platelets	197.200 ± 129,264 ^a^	253.800 ± 133,391 ^a^	226.466 ± 138,153 ^a^	181.600 ± 133,605 ^a^	220.400 ± 62,247 ^a^	319.400 ± 203,191 ^a^
Leukocytes	10.200 ± 4423 ^a^	10.200 ± 6398 ^a^	12.512 ± 5570 ^a^	5.680 ± 2612 ^a^	7.998 ± 2709 ^a^	9.580 ± 8764 ^a^
Neutrophils	7.225 ± 3922 ^a^	5.839 ± 6935 ^a^	9.700 ± 7246 ^a^	2.479 ± 1470 ^a^	4.034 ± 3036 ^a^	5.925 ± 8030 ^a^
Lymphocytes	1.892 ± 662 ^a^	2.930 ± 1495 ^a^	2.026 ± 1369 ^a^	2.157 ± 1763 ^a^	3.015 ± 1069 ^a^	2.866 ± 821 ^a^
Monocytes	401.20 ± 88.06 ^a^	561.60 ± 257.29 ^a^	695.40 ± 448.91 ^a^	359.00 ± 127.63 ^a^	810.40 ± 37.91 ± 340.62 ^a^	533.73 ± 531.56 ^a^
Eosinophils	680.80 ± 692.92 ^a^	688.80 ± 758.70 ^a^	122.60 ± 75.49 ^a^	684.60 ± 864.62 ^a^	50.40 ± 77.87 ^a^	255.40 ± 237.46 ^a^
ALT	32.14 ± 10.97 ^a^	99.86 ± 99.19 ^a^	54.81 ± 63.25	31.50 ± 6.81 ^a^	72.98 ± 63.39 ^a^	36.46 ± 26.12 ^a^
Creatinine	1.00 ± 0.21 ^a^	0.96 ± 0.28 ^a^	0.92 ± 0.33 ^a^	0.98 ± 0.13 ^a^	0.92 ± 0.08 ^a^	2.12 ± 2.45 ^a^
Alkaline phosphatase	51.82 ± 37.91 ^a^	58.86 ± 52.13 ^a^	28.20 ± 11.75 ^a^	49.80 ± 25.55 ^a^	89.50 ± 81.69 ^a^	55.48 ± 23.05 ^a^
Urea	32.00 ± 7.11 ^a^	48.00 ± 32.18 ^a^	36.60 ± 9.86 ^a^	27.80 ± 8.23 ^a^	31.40 ± 12.38 ^a^	72.21 ± 88.55 ^a^

Reference values: Erythrocytes 5.50–8.00 (mm^3^), Hemoglobin 12.00–18.00 (g/dL), Hematocrit 37.00–55.00 (%), Platelets 200,000–500,000 (mm^3^), Leukocytes 6000–17,000 (mm^3^), Neutrophils 3600–13,090 (mm^3^), Lymphocytes 720–5100 (mm^3^), Monocytes 180–1700 (mm^3^), Eosinophils 120–1700 (mm^3^), ALT 10–88 (U/L), Creatinine 0.5–1.5 (mg/dL), Alkaline phosphatase 21–60 (U/L), Urea 20–156 (g/dL). Source: [23]. ^a^—Lowercase letter indicates no significant difference within the row.

## Data Availability

The data presented in this study are available on reasonable request from the corresponding author.

## Abbreviations

The following abbreviations are used in this manuscript:

TVT	Transmissible Venereal Tumor
CAAF	Fine-needle aspiration cytology
CEUA	Animal Ethics Committee
CG	Control group
AHTm	Minor autohemotherapy associated with vincristine sulfate
AHTmO_3_	Ozonated minor autohemotherapy associated with vincristine sulfate
WT	Weekly time point
ALT	Alanine aminotransferase
